# Grip Force Is Part of the Semantic Representation of Manual Action Verbs

**DOI:** 10.1371/journal.pone.0009728

**Published:** 2010-03-16

**Authors:** Victor Frak, Tatjana Nazir, Michel Goyette, Henri Cohen, Marc Jeannerod

**Affiliations:** 1 Département de kinanthropologie, Université du Québec à Montréal (UQÀM), Montréal, Québec, Canada; 2 Institut de Réadaptation Gingras-Lindsay de Montréal, Centre de recherche interdisciplinaire en réadaptation du Montréal métropolitain, Université de Montréal, Montréal, Québec, Canada; 3 CNRS UMR 5015, Institut des Sciences Cognitives, Bron, France; 4 CNRS UMR 8189, Université Paris Descartes, Boulogne-Billancourt, France; The University of Western Ontario, Canada

## Abstract

Motor actions and action verbs activate similar cortical brain regions. A functional interference can be taken as evidence that there is a parallel treatment of these two types of information and would argue for the biological grounding of language in action. A novel approach examining the relationship between language and grip force is presented. With eyes closed and arm extended, subjects listened to words relating (verbs) or not relating (nouns) to a manual action while holding a cylinder with an integrated force sensor. There was a change in grip force when subjects heard verbs that related to manual action. Grip force increased from about 100 ms following the verb presentation, peaked at 380 ms and fell abruptly after 400 ms, signalling a possible inhibition of the motor simulation evoked by these words. These observations reveal the intimate relationship that exists between language and grasp and show that it is possible to elucidate online new aspects of sensorimotor interaction.

## Introduction

The consequence of lesions and the functional overlap between language and motor action strongly suggest that aspects of language and action are intimately linked. In early writings on apraxia, Liepmann [1] described patients unable to carry out voluntary and skillful movements following verbal requests with their left body parts following lesions to the forebrain. He named this condition *sympathetic apraxia*. For Geschwind [2], this syndrome followed the interruption or blocking of information transfer between the language and motor brain areas. Also, in the case of articulatory dyspraxia, with difficulties in speaking or pronunciation, the question remains as to whether it is a disorder of motor control or an expression of aphasia constrained by syntactic categories. Broca [3], in his original presentation of patient Tan, already presented evidence of articulatory disturbance in speech (aphemia) as a result of frontal cerebral damage.

Action verbs and motor actions activate similar cortical brain areas [4] [5]. An increasing number of studies have shown that the sensorimotor components of word meaning activate cortical regions overlapping with the neural systems involved in the perception and execution of actions described by the words. For example, processing verbally presented actions activates corresponding sectors of the motor system, depending on the effector (hand or foot) used in the listened-to action [6] [7]. It is also known that reading the word *write* activates the cortical motor areas involved in moving the hand [8]. Moreover, in sign language there is a close semantic relationship between the gestures and the function of the object expressed (e.g., hammer or scissors in American Sign Language), suggesting that transmodal processes are implicated in the semantic representations [9]. In addition, lesion evidence also suggests that both language and pantomime of object use are affected in patients with left brain damage [10]. These studies and numerous observations strongly suggest that the brain areas subtending object-oriented actions are closely related to the brain areas involved with language [11].

Glenberg [12] has proposed that linguistic meaning is grounded in bodily activity when we are engaged in action that carries into effect [see also, 13]. In this perspective, the linguistic message is functionally assimilated in the intention of action [14]. It has been proposed that if intention of a motor action were to be extended in time it would progressively turn into a motor simulation of that action [15]. This simulation of action becomes, according to Prinz [16], an integral part of the sensorimotor interface. Over the course of a given action, intention and simulation may become indistinguishable. In the same vein, it has been suggested that intention provides the cement binding afferent stimulation and efferent response. For Hommel et al. [17] these action plans are motor images. For Jeannerod [15], intentionality is at the core of the representation of action, as incoming information modulates ongoing action. In this view, intention and simulation are the unifying elements between the linguistic stimulus and the action response.

It has recently been suggested that there is a lexical-semantic competition that interferes with the action, once an action is triggered. Boulenger et al. [18] and Nazir et al. [19] have proposed an experimental design for the investigation of language-kinematics interaction. In their experiments, a manual action verb is presented visually at varying moments during the execution of ballistic gestures (i.e., 0 ms, 50 ms, 200 ms following movement onset) and the transport speed of the wrist is measured. The action is generally perturbed when the action verb is presented once a movement is initiated, testify to a complex interaction between the linguistic and muscle components of action. However, in this experimental paradigm the perturbation is not time-locked to verb onset because the characteristics of the movement itself (i.e., the ballistic momentum) partially mask the immediate impact of the linguistic stimulus. Information about when exactly word processing starts to affect motor behavior is therefore not available.

We propose a novel approach that will allow *online* examination of the relation between language and action. By analyzing modulations of the precision grasp of a cylinder with an integrated force sensor, we shall examine the extent to which listening to words related to the action of the prehensile hand can affect grip force. It is known that Broca's area is activated during the simulation of grasp movements [20] [21]. However, the relationship between language and grip force has not yet been investigated. The approach used in the present study will help determine when word processing influences motor behavior. Force variations in prehensile grip, while listening to manual action and control words, were analysed in order to consider the links between the kinematics of the hand and linguistic content. The question of the relationship between language and action has been with us for over a century. The convergence of efforts in the elucidation of this question has been to determine how the two interact. Whatever the methodology (ERP, fMRI, behavioral, and now grip force studies), all work in this area has attempted to understand the influence of one of the other in as small a time window as possible. ERP methods come closest and now, with our grip force paradigm, we suggest a complementary alternative that will provide, we hope, a new way to look at and better understand the nature of the relationship.

## Methods

### Ethics Statement

Ethics approval was obtained from the ethics committee of Montreal Centre for Interdisciplinary Research in Rehabilitation.

### Participants

Six monolingual French native volunteers, 2 men and 4 women (age range: 15–52; median age was 23 years) participated in this study. All were right- handed as assessed with the Edinburgh Handedness Inventory [22]. All subjects (or parents in the case of the youngest subject) gave written consent to be included in the study. None of the authors participated as subjects in the study.

### Stimuli

A total of 35 nouns and 35 verbs, controlled for frequency, number of letters, number of syllables, bi- and trigram frequency [23] served as stimuli. All verbs denoted actions performed with the hand or arm (e.g., write, throw) while nouns referred to imaginable concrete entities without specific motor associations (e.g., mill, cliff) and were used as control words. Words that could be used as both nouns and verbs were excluded from the selection. Words were spoken by an adult male and recorded on a digital voice recorder (Olympus DS-50), in two consecutive sessions with a pause of five minutes between the sessions. All 35 verbs were recorded at one session, and the 35 nouns were recorded in the same manner, at a separate recording session. The resulting two recordings were transferred to a computer and each word in the two lists was individually extracted and saved to a file. Comparison of voice amplitude of the words in the two lists (nouns, verbs) yielded no statistically significant difference.

Digitized lists of words were then generated from the 70 items. Within these lists, one randomly selected target word (noun or verb) was repeated 17 times while all remaining words were presented only once. Participants thus listened to a total of 86 items. Mean word duration was 684 ms and there was an interval of 1000 ms between word presentations. Word order was randomized between subjects.

### Procedure

**Figure 1:**
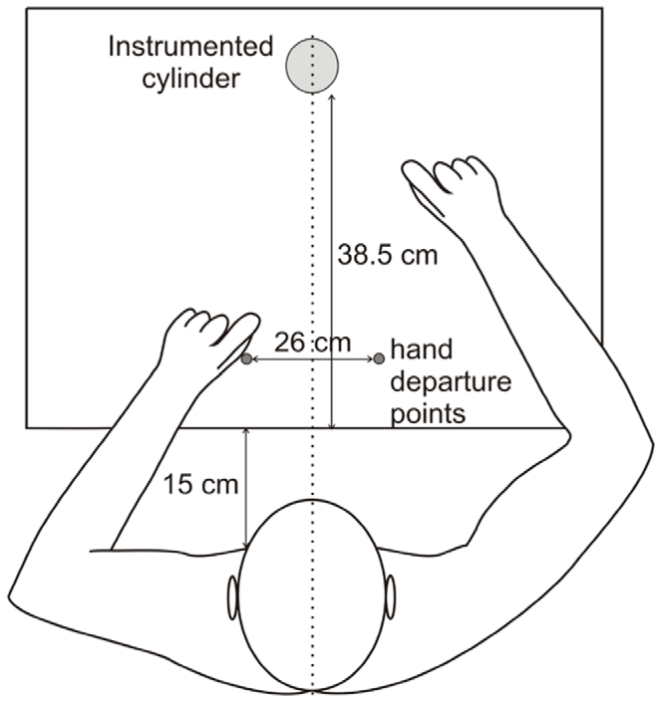
The experimental paradigm. Participants wore headphones and were seated on a chair facing a table. The instrumented cylinder was placed at a distance of 53.5 cm from their chest.

**Figure 2:**
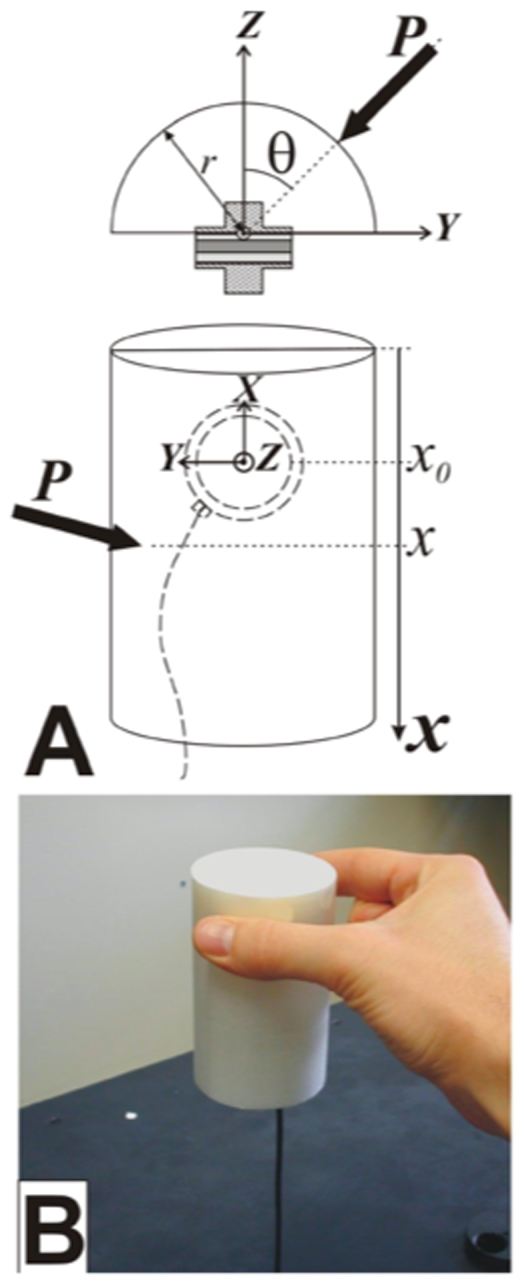
Functioning of the instrumented cylinder. **A.** The apparatus is designed to measure the orientation (θ) and vertical location (*x*) of the applied force (*P*) by either the index or thumb while exerting a grip force. These parameters are computed from outputs of a F/T sensor (with axes *X*, *Y* and *Z*) embedded in the two half-cylinders using two T-adaptors. **B**. The participants were asked to lift the cylinder with the thumb and index fingers of the right hand and hold it at about 5 cm above the table.

Participants wore headphones and were seated on a chair without armrests, facing a table on which the instrumented cylinder was placed at a distance of 53.5 cm from their chest (Figure 1). The cylinder weight was 267 g. Participants were first asked to rest both hands on the table and touch a home pad with their thumbs (5 cm from the edge of the table and 13 cm to either side of the midline). They were then asked to lift the cylinder [Figure 2A; for a technical description of the apparatus, see [24]] with the thumb and index finger of the right hand and hold it at about 5 cm above the table (Figure 2B). We used a cylindrical object, so there was no imposed grasp orientation. The participants maintained this position by flexing the shoulder while keeping the elbow in full extension. Participants listened to the list of words and silently counted the occurrence of the target word while performing this motor task. The target word was an action verb in one condition, and a noun in the other.

Each subject participated in two sessions, counterbalanced across subjects. They were instructed to listen to and count the occurrence of the target words . Thus, subjects were presented with 35 nouns and 51 verbs (one of the verbs was repeated 16 times) in one session, and they were presented with 35 verbs and 51 nouns in the other session. Verb and noun list presentations were randomized across subjects. . If the first session had a target verb, then the second one had a noun as a target, and conversely . Subjects kept their eyes closed for the duration of the experiment. At the end of each session, the cylinder was lowered on the table and the participants were asked to give the number of times the target word was presented.

### Data Acquisition

The output of three force and three moment signals (Fx, Fy, Fz, Mx, My, Mz) captured with the cylinder was generated by a standalone F/T sensor system controller (ATI Industrial Automation, NC, USA). Fx is the longitudinal force exerted on the cylinder, Fy and Fz are the radial and compression forces, respectively (Figure 2A). Mx, My, and Mz are the moments. The signals were recorded with an AT-MIO-16E-10 A/D card (National Instruments, TX, USA) and acquired at 100 Hz per channel, for about 145 seconds. The list of digitized words was delivered through a D/A channel of another AT-MIO-16E-10 card connected to the headphones. Both cards were synchronized such that the output of the digitized list of words automatically triggered the acquisition of grip information.

### Data analysis

Prior to data analysis, each signal component was filtered at 10 Hz with a fourth-order, zero-phase, low-pass Butterworth filter. The grip force was computed by taking the resultant force of Fx, Fy and Fz. Data were then segmented from the onset of one word to the onset of the following word. Since the level of force applied on the cylinder differed between subjects, each segment of the signal amplitude was normalized by subtracting the lowest point value and dividing the result by the span range (max – min value), thus yielding values ranging between 0 and 1. Normalized signals for nouns, verbs and target words were averaged for each participant and the grand mean was computed for each condition. As the number of target words was smaller than that of nontargets (17 vs. 34), a random selection of 17 nontarget words were extracted from each condition to be used in the data analyses. In order to determine whether the vertical load (gravitational and inertial) of the cylinder did influence the results in any way, analyses were conducted with and without the vertical force component (Fx) . Comparisons on the grip force normalized curves were also run. Two curves were generated: one taking into account computations of all axes (Fx, Fy, Fz); and another with the forces orthogonal to the cylinder (Fy, Fz). The statistical comparison of the two curves yielded R2 = 0.9996, showing that the load charge had no effect on the curves. This was an expected result since the analysis was conducted on the variation of force in the system, and the cylinder is considered to be in a quasi-static state — thus contributing very little effect, if any.

## Results

**Figure 3:**
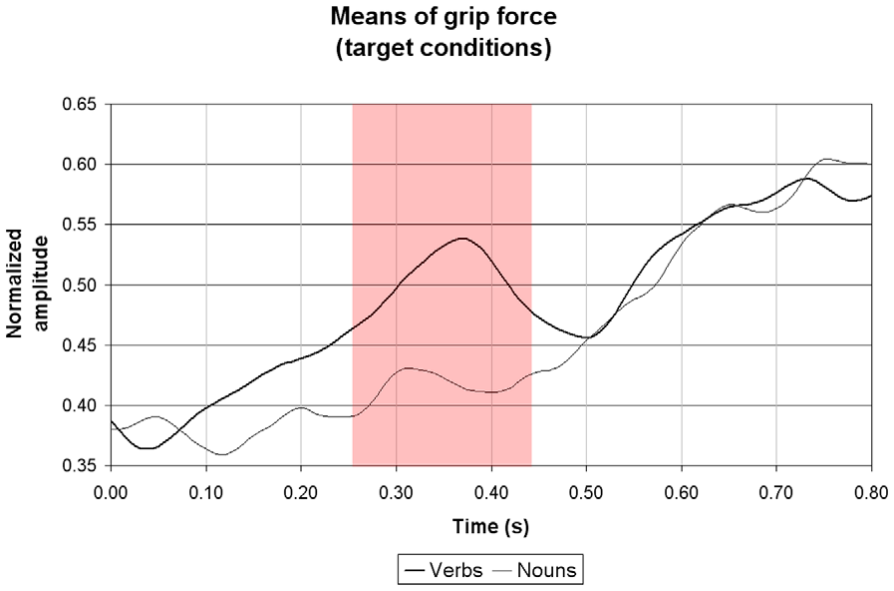
Normalized grip force amplitude. Grand average of normalized grip force amplitude of verbs and nouns –when they are targets– between the onset of a stimulus word until the end of the longest word duration. Compared to the nouns, the presence of verbs induced an early change in the signal following word onset. The shaded portion of the graph, starting at 260 ms and ending at 430 ms, shows the area where the two curves are significantly different (p<0.05).

Figure 3 displays the grand mean of normalized grip force amplitude of verb and noun signals between the onset of a stimulus word until 800 ms later, corresponding to about the end of the longest word duration. There was a change in grip force when the target word was a verb , but not when it was a noun An increase in force was observed at about 100 ms following the verb display, deviated significantly from the noun curve at approximately 260 ms and fell abruptly after reaching a peak at 380 ms. A ms-by-ms paired t-test was conducted on the data points defining both curves. A significant difference was noted between 260 and 430 ms (p<0.05). Analyses of non-target verbs and nouns showed no significant difference in grip force.

## Discussion

The aim of this study was to investigate the interaction between the motor representation of manual action verbs and linguistic content online. Until now, it was not clear when the processing of linguistic information (i.e., verbs) influenced motor behavior. The present results indicate that it is possible to determine through online analysis of grip force modulation when this effect occurs.

Reading manual action verbs perturbs reaching movements [18] [19]. Reaching and grasping are intimately linked [25] and it is likely that manual action verbs can impact upon grasping action. Reaching implicates proximal muscle systems under the control of the two cerebral hemispheres. Grasping with the preferred right hand implicates distal muscles under — as is the case for most of the verbal system — left hemispheric control.

A number of interpretations can be offered for the fact that the processing of verbs and processing of the corresponding actions share similar brain resources. A first possibility is that a verb activates cerebral motor areas since it brings about a motor image of the verbally presented action — suggesting that activation of the motor system takes place at the post-lexical level. The motor simulation thus provides the pragmatic knowledge congruent with the underlying action and complements the semantic recognition of the verb.

Pulvermüller et al. [26] had proposed a contrasting view of the motor activation induced by verbs: activation is not the consequence of the relationship between the verbs and the simulated actions but rather that it is inherently linked with lexical-semantic processing. A key argument for this interpretation is that the activation of the motor system occurs early in the course of presentation of the verb (under 200 ms following onset of display).

The results of the present study indicate that these two views can actually be integrated along a motor continuum of linguistic information. The observed increase in grip force occurring with the presentation of verbs can be interpreted as both the progression of the spontaneous muscular facilitation evoked by the verb during lexical-semantic processing [27] and as the incomplete inhibition of the motor output during simulation [15] [28]. To our knowledge, this is the first time that a demonstration of this phenomenon is made, indicating that the structures that participate in the retrieval of verbs also partake in the control of motor behavior. Thus, simulation of action is at the interface between verb comprehension and motor production [16]. This is a difficult issue to resolve, as a number of authors have interpreted this facilitation effect as an incomplete inhibition of muscular activity [28]. The present results can also be taken as evidence for a facilitation mechanism of the lexical semantic treatment and an incomplete central inhibitory mechanism, as reflected in the decrease of grip strength.

It is important to note that the variations in force level were subliminal as subjects did not report, even when specifically questioned at the end of the experiment, that they were aware of observable changes in grasp force between the different experimental conditions. This suggests that onset of linguistic information can generate motor simulations, producing peripheral muscle changes that are not under conscious control or awareness.

The crosstalk between language processes and overt motor behavior provides unambiguous evidence that verbs and motor action share common cortical representations, suggesting that cortical motor regions are indeed involved in verb retrieval. As this happens during a manual action, such as holding an object with a precision grasp, it also means that the muscular changes related to the simulation and the action, although closely tied, constitute separable elements. This distinction has been reported following damage to frontal brain areas [29]. Furthermore, hemiplegic patients are capable of simulating manual actions even though they are paralyzed as a result of brain injuries in M1 [30]. The approach presented here opens up a new avenue of research investigating the impact of complex language and speech activity in healthy subjects and clinical populations with movement or language disorders.
